# Two new species of *Feroperis* Lafer (Carabidae, *Pterostichus*) from China, with a key to all known Chinese species in this subgenus

**DOI:** 10.3897/zookeys.799.28834

**Published:** 2018-11-28

**Authors:** Xiaojie Sun, Hongliang Shi, Weiguo Sang, Jan Christoph Axmacher

**Affiliations:** 1 College of Life and Environment Sciences, Minzu University of China, Beijing 100081, China Minzu University of China Beijing China; 2 College of Forestry, Beijing Forestry University, Beijing 100081, China Beijing Forestry University Beijing China; 3 UCL Department of Geography, University College London, London WC1E 6BT, UK University College London London United Kingdom

**Keywords:** China, *
Feroperis
*, ground beetles, morphology, taxonomy, temperate forest

## Abstract

Two new *Pterostichus* species (Coleoptera, Carabidae) in the subgenus Feroperis Lafer, 1979 are described from Zhangguangcai Mountain, northeastern China: Pterostichus (Feroperis) silvestris Sun & Shi, **sp. n.** and Pterostichus (Feroperis) maryseae Sun & Shi, **sp. n.** Detailed descriptions and illustrations of the male endophallus and female reproductive tracts for these new species are provided, along with a key to the five known species of the subgenus in China.

## Introduction

The subgenus Feroperis in the genus *Pterostichus* Bonelli, 1810, was erected by Lafer (1979) and originally comprised 17 species. Subsequently, eight species were added to this subgenus (Park and Kwon 1996, Berlov and Berlov 1996, Lafer 2011), all of them now valid. Not long ago, three of the species originally placed by Lafer (1979) into *Feroperis* were downgraded to subspecies rank (Sundukov 2013). Thus, prior to this study, the subgenus Feroperis Lafer included 22 species and three subspecies (Bousquet 2017). After its establishment, *Feroperis* has been regarded as synonym of other subgenera of *Pterostichus* by some authors. For example, Kryzhanovskij et al. (1995) placed it as a synonym of *Petrophilus* Chaudoir, 1838. Based on a preliminary phylogenetic analysis on characteristics of the endophallus, Sasakawa and Kubota (2006) confirmed, too, the synonymy of *Feroperis* with *Petrophilus*. Most recently, Sundukov (2013) treated the subgenus in question as a synonym of *Morphnosoma*. In the present work, we follow the dominant view (e.g. Park and Kwon 1996, Lafer 2011) and catalogues (Lorenz 2005, Bousquet 2017), where Feroperis is generally regarded as a valid subgenus of Pterostichus.

Members of the subgenus Feroperis can be distinguished from other subgenera within *Pterostichus* by the following combination of characters: ridge between outer basal foveal groove of pronotum and lateral margin strongly carinate; third elytral interval with three or more setigerous pores which are positioned adjacent to the second stria; fifth elytral interval without setigerous pores; length of metepisternum shorter than its basal width; metatrochanter without seta; fifth tarsomere setose beneath; males without sex-specific differentiation of sternum VI or VII; median lobe of aedeagus with both apical orifice placed on the dorsal surface and apical lamella simple or thickened; right paramere thick, more or less elongate and with apex bent and pointed.

Most species of *Feroperis* hitherto known were recorded from the Russian Far East (15 spp.) and the Korean Peninsula (8 spp.). Only three species: *P.acutidens* (Fairmaire, 1889), *P.melanodes* (Chaudoir, 1878), and *P.rasilis* Park & Kwon, 1996 have been reported from China (Bousquet 2017, Lafer 2011). Compared to the rich fauna in neighboring countries, the number of known species of *Feroperis* in China is conspicuous low, implying a high likelihood of new species to be discovered. Over the last decade, our investigations of forest ground beetle communities in China’s regenerating temperate forest landscapes resulted in collections of large numbers of *Feroperis* specimens. This material includes samples from the forests of the Zhangguangcai Mountain, which is situated on the border between Heilongjiang Province and Jilin Province, northeastern China. After detailed studies of these specimens, we ascertained that they belong to two species that are new to science. Hence, in this paper, we are describing and illustrating these two new species, and providing a key to all known Chinese species of the subgenus Feroperis.

## Methods

A total of 80 pitfall traps were placed at five distinct forest types representing mature, secondary and planted forests, on the Zhangguangcai Mountain range at elevations between 771 and 985 m. All carabid beetles collected in the traps were subsequently pinned and dissected using routine techniques (Shi et al. 2013). All the examined specimens are housed in the collections of the Institute of Zoology, Chinese Academy of Science, Beijing, China (IZAS).

Body length of specimens was measured from the anterior margin of the labrum to the apex of the elytral suture; maximal width of head (HW) was recorded as the greatest width between the lateral margins of the eyes; PW and EW represent the greatest widths of the pronotum and elytra, respectively; the apical width of pronotum was measured as the width between the tips of pronotal anterior angles; basal width of the pronotum (PB) was recorded as the width between the posterior angles of the pronotum; the pronotum length (PLm) was the respective length along the midline of the pronotum; the total pronotum length (PLt) was the length between the anterior angles and posterior angles of the pronotum; the length of elytra (EL) was measured from the apex of the scutellum to the sutural apex. All measurements were made with the aid of an ocular micrometer under a Nikon SMZ18 stereomicroscope.

Male genitalia were extracted using forceps, and endophalli were prepared for 15 and 21 specimens of the two species, respectively, by microinjection. The median lobe of the aedeagus was soaked in 10% KOH solution at room temperature for 8–20 hours and subsequently stored in 100% ethanol. The basal orifice of the aedeagus was injected with 100% ethanol with a microinjector to fully evert the endophallus. The treated male genitalia were kept in 100% ethanol during the initial investigations, and they were later transferred into glycerol for permanent storage. Female genitalia were prepared from the last one or two abdominal ventrites of 9 and 12 specimens representing the two species, respectively, and immersed in 10% KOH solution at room temperature for 8–20 hours. The female genitalia were then extracted from the abdominal segments and stained in Chlorozol Black E-saturated solution based on 70% ethanol for approximately 10 seconds, before being rinsed and stored in 70% ethanol for imaging and permanent storage. The species examination and subsequent descriptions were made using a Nikon SMZ18 binocular stereoscope, while a Leica205C stereomicroscope equipped with photographic adapters was used to take images of the specimens and their genitalia.

## Results

### Subgenus Feroperis

Taxon classificationAnimaliaColeopteraCarabidae

Lafer, 1979

Feroperis Lafer, 1979: 5. Type species: Feroniajugens Tschitschérine, 1893, by original designation (Lafer 1979).

#### Diagnosis.

Body of medium size (13.0–17.5 mm), surface completely black and polished, palpi brown, legs and antennae black or dark brown. Head with very weak punctures; eyes large and convex; antennae reaching the base of pronotum. Pronotum more or less round, 1.3–1.6 times wider than the head; anterior angles rounded, moderately or strongly protruding; lateral border gradually widened and then narrowed towards the base, maximum width near anterior third; lateral channel narrow in front but expanding towards the base in the posterior half; basal margin slightly concave in the middle, either rectilinear or obliqued on the sides; basal foveae usually slightly punctate, outer basal foveal groove deep, reaching the basal margin, the inner basal foveal groove shallower and not reaching the basal margin; carinae between lateral margin and outer basal foveae clearly expressed, perpendicular or inclined towards basal margin, well-separated from the lateral channel and approaching to it near the basal margin; pronotum with two lateral setae, one at the posterior angle, the other one near the pronotal maximum width; pronotum disc with transverse wrinkles. Elytra wide, 1.10–1.35 times wider than pronotum, 1.5–1.6 times wider than elytral width; lateral margins subparallel, widest in the middle; basal ridge continuous, forming an indistinct obtuse angle with the elytral lateral margin; humeral teeth small but distinct; striae deeply incised, without or with very fine punctures; intervals moderately convex; parascutellar pore present; scutellar striae present; third interval with three or more setigerous pores, usually 3–4, rarely 5–6, usually adjacent to the second striae; seventh interval with two preapical pores; umbilical setigerous series on the ninth interval, interrupted in the middle. Hind wings very small, not functional. Metepisternum short, its length along inner margin subequal to the width of anterior margin; sterna IV–VI with a pair of central setae; males with one pair, females with two pairs of marginal setae on sternum VII, slightly removed from the apical margin. Mesofemora and metafemora with two setae on posterior margin; metatrochanters without setae; metacoxae with two setae; fifth tarsomeres setose beneath. Median lobe of aedeagus slender, bent usually at about 90 degrees; median lobe almost straight in median portion (between the basal bend and apical lamella); apical orifice opened to the dorsal-left side; the shape of the apical portion of apical lamella shows species-specific differences. Stylomere 2 with two ensiform setae at the outer margin and one ensiform seta in the middle part of its inner margin; two nematiform setae in a short fovea near the apex of inner surface (i.e. Lafer 1979).

### Key to Chinese species of subgenus Feroperis Lafer

**Table:** 

1	Posterior angles of the pronotum obtuse or weakly protruding, lateral border not or only slightly thickened at the posterior angles (Fig. 7A–B)	**2**
–	Posterior angles of pronotum strongly protruding forming strong denticles, lateral border strongly thickened at the posterior denticles (Fig. 7C–D)	**3**
2	Pronotal posterior angles obtuse, not forming denticles; carinae between basal foveae and lateral margins shallower; male genitalia unknown (Fig. 14B)	***P.melanodes* (Chaudoir, 1878)**
–	Pronotal posterior angles weakly protruding, forming small denticles; carinae between basal foveae and lateral margins stronger; apical lamella of aedeagus widened to apex; length approximate 1.5 times as its basal width (Figs 1–2)	***P.silvestris* sp.n.**
3	Apical lamella of aedeagus distinctly widened to apex, length approximate 1.5 times as basal width (Park and Kwon 1996: fig. 2F–G)	***P.rasilis* Park & Kwon, 1996**
–	Apical lamella of aedeagus gradually narrowed to apex, length 1.0–1.2 times as basal width	**4**
4	Pronotal posterior angles with smaller denticles (Fig. 7C–D); in lateral view, ventral margin of apical lamella of aedeagus straight before apex (Fig. 9C); apical lamella apex slightly truncate in dorsal view (Fig. 9A)	***P.maryseae* sp.n.**
–	Pronotal posterior angles with larger denticles (Fig. 14A); in lateral view, ventral margin of apical lamella of aedeagus slightly curved before apex; apical lamella apex rounded in dorsal view (Lafer 1979: figs 1–3)	***P.acutidens* (Fairmaire, 1889)**

### Pterostichus (Feroperis) silvestris

Taxon classificationAnimaliaColeopteraCarabidae

Sun & Shi sp. n.

http://zoobank.org/FE776971-524A-4E86-A73C-FF0AEE663226

Figs 1–3 , 4–6

#### Type locality.

CHINA: Heilongjiang Province, Hailin County, Taipinggou Forest Farm (44°24.6168'N, 128°24.5570'E), altitude 985 m.

#### Type materials.

**Holotype** (IZAS): male, body length 12.9 mm, board mounted, genitalia dissected and glued on plastic film pinned under specimen, “China, Heilongjiang / Taipinggou Forest Farm / Zhangguangcai Mountain”; “Pitfall trap, 985 m, 2016.VI.08 / 44°24.6168'N, 128°24.5570'E / Diekman, MZUC”; “HOLOTYPE ♂ / *Pterostichus* (*Feroperis*) / *silvestris* sp. n. / des. SUN & SHI 2018” [red label]. Paratypes (a total of 1643 specimens [906 males and 737 females], all in IZAS): 247 males and 132 females, the same data as holotype, but labeled as paratype. 126 males and 227 females, “China, Heilongjiang / Taipinggou Forest Farm / Zhangguangcai Mountain”; “Pitfall trap, 985 m, 2016.VI.21 / 44°24.6168'N, 128°24.5570'E / Sun Xiaojie, MZUC”; “PARATYPE / *Pterostichus* (*Feroperis*) / *silvestris* sp. n. / des. SUN & SHI 2018” [red label]. 284 males and 160 females, “China, Heilongjiang / Taipinggou Forest Farm / Zhangguangcai Mountain”; “Pitfall trap, 985 m, 2016.VII.05 / 44° 24.6168'N, 128°24.5570'E / Sun Xiaojie, MZUC”; “PARATYPE / *Pterostichus* (*Feroperis*) / *silvestris* sp. n. / des. SUN & SHI 2018” [red label]. 192 males and 156 females, “China, Heilongjiang / Taipinggou Forest Farm / Zhangguangcai Mountain”; “Pitfall trap, 985 m, 2016.VIII.03 / 44°24.6168'N, 128°24.5570'E / Sun Xiaojie, MZUC”; “PARATYPE / *Pterostichus* (*Feroperis*) / *silvestris* sp. n. / des. SUN & SHI 2018” [red label]. 57 males and 62 females, “China, Heilongjiang / Taipinggou Forest Farm / Zhangguangcai Mountain”; “Pitfall trap, 985 m, 2016.VIII.31 / 44°24.6168'N, 128°24.5570'E / Sun Xiaojie, MZUC”; “PARATYPE / *Pterostichus* (*Feroperis*) / *silvestris* sp. n. / des. SUN & SHI 2018” [red label].

#### Diagnosis.

This new species can be distinguished from all other species of the subgenus by the combination of following characters: (1) lateral margins of pronotum evenly convex at about anterior two thirds, then gradually contracted and almost straight before posterior angles; (2) pronotum posterior angles weakly protruding, forming indistinct denticles, lateral border not widened at posterior angles, its width subequal to the lateral border of pronotum; (3) apical lamella of the aedeagus as long as 1.5 times its basal width; apex capitate in dorsal view, widened at both left and right margins, but only slightly thickened in lateral view; apical lamella distinctly oblique to the right in dorsal view, with ventral margin slightly twisted dorsally in lateral view (Fig. 2A).

The new species is special in the subgenus for its male genitalia with apical lamella capitate, both margins widened near apex in dorsal view, and not strongly thickened in lateral view. These aedeagal characters can distinguish it from most species of *Feroperis* except for the five species *P.chechcirensis* Lafer, 1979, *P.vladivostokensis* Lafer, 1979, *P.rasilis* Park & Kwon, 1996, *P.seungmoi* Park & Kwon, 1996, and *P.pawlowskii* Lafer, 2011. Among them, *P.chechcirensis* can be readily distinguished by its pronotal posterior angles being obtuse, without any trace of denticle; *P.vladivostokensis* and *P.rasilis* are different in their pronotal posterior angles being strongly pointed, forming very large denticles. From the remaining two species, *P.seungmoi* can be easily identified by its characters that widest of pronotal lateral channels at about 1/3 length to the posterior margin, so *P.silvestris* is considered to be closer to *P.pawlowskii* (North Korea: Nampotesan, 41°44'N, 128°24'E), based on their more similar external characteristics and close areas of distributions.

Considering *P.pawlowskii*, the new species is distinguishable from it by the presence of micro-punctures on vertex and by the pronotal disc with very fine punctures rather than reticular traces. Furthermore, these two species can be also distinguished by: (1) the pronotum widest at about basal 2/3 in *P.pawlowskii*, vs widest at about 3/5 in *P.silvestris*; (2) in *P.silvestris*, the pronotum less constricted to the base; (3) in *P.pawlowskii*, the apical lamella of aedeagus shorter, its length reaching as long as 1.2 times the basal width, apex almost straight or very weakly bent to the left in dorsal view; while in *P.silvestris*, the apical lamella of the aedeagus is distinctly longer, its length about 1.5 times the basal width, apex distinctly bent to the right in dorsal view.

**Figures 1–3.**
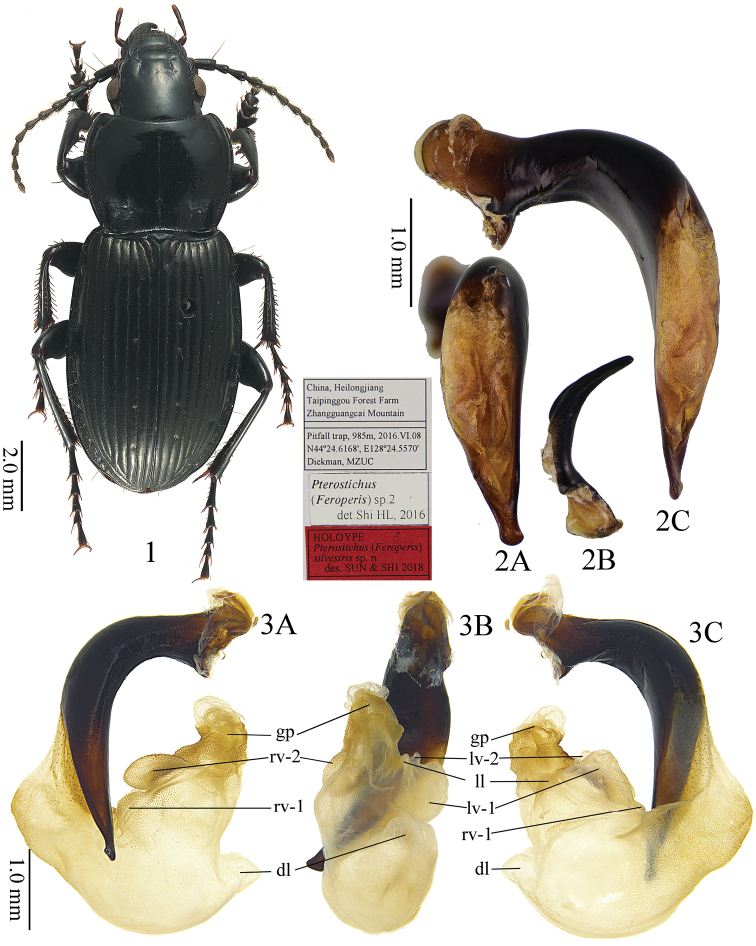
Pterostichus (Feroperis) silvestris sp. n. **1** Habitus and labels of holotype **2** Male genitalia of holotype **A** dorsal view of median lobe, **B** right paramere, **C** left lateral view of median lobe **3** Endophallus of a paratype **A** right lateral view, **B** ventral view, **C** left lateral view.

#### Description.

**Figures 4–6.**
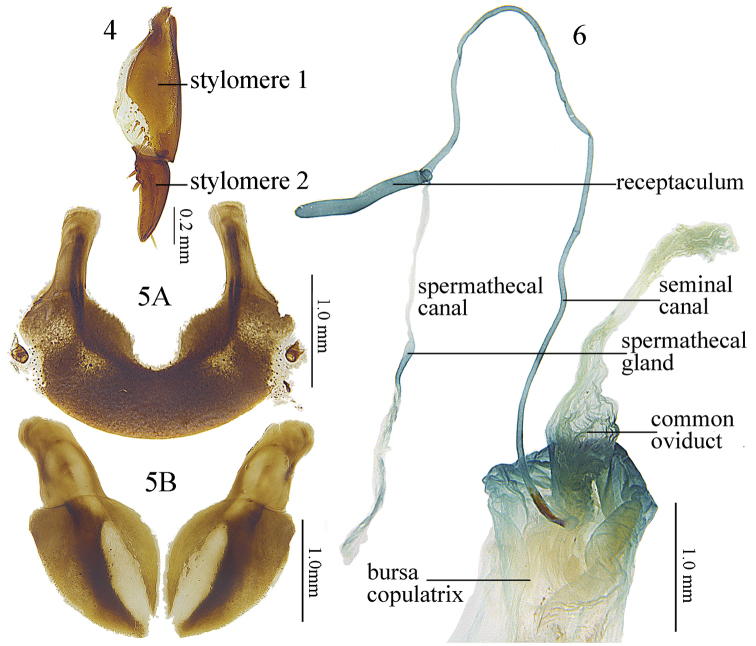
Pterostichus (Feroperis) silvestris sp. n., a female paratype **4** Stylomere of female ovipositor, ventral view **5 A** tergum VIII, **B** sternum VIII **6** Female reproductive tracts.

**Figure 7.**
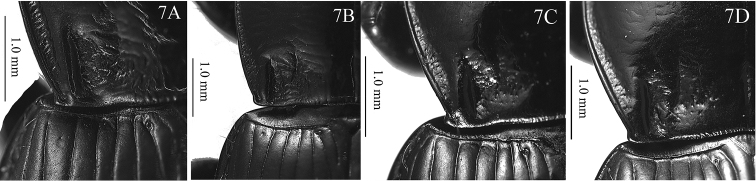
Pronotum left posterior angle and basal area of Pterostichus (Feroperis) spp. n. **A–B***P.silvestris***C–D***P.maryseae*.

Body length 12.8–14.9 mm (mean ± SD: 13.8 ± 0.65, *n* = 20), both sexes with similar body shape. Dorsal surface black and shiny; tarsi and antennae dark brown; head and pronotum without microsculpture; elytra with very fine and isodiametric microsculpture. **Head** mostly smooth, polished, with very fine and sparse punctures on vertex and occiput; eyes moderately convex; antennae reaching the base of pronotum. **Pronotum** wider than the head (PW/HW =1.11–1.52, mean ± SD: 1.44 ± 0.09, *n* = 20); rounded in shape, widest at about 3/5 length to the posterior margin (PW/PLt = 1.11–1.38, mean ± SD: 1.27 ± 0.07, *n* = 20; PW/PLm = 1.22–1.52, mean ± SD: 1.40 ± 0.07, *n* = 20); lateral margins evenly convex from apex to about basal 1/3, then gradually contracted and almost straight before the posterior angles (PW/PA = 1.27–1.47, mean ± SD: 1.37 ± 0.05, *n* = 20; PW/PB = 1.23–1.43, mean ± SD: 1.32 ± 0.05, *n* = 20); apical width of pronotum nearly same as its basal width (PB/PA = 0.94–1.14, mean ± SD: 1.04 ± 0.05, *n* = 20). Anterior angles obtuse and rounded, distinctly contracted inward; lateral channels narrow in front of midpoint and gradually expanded towards the base, with flatten and sparse punctures on them. Posterior angles obtuse, weakly protruding and forming weak denticles of angle exceeding to 100° (147.4°–166.8°, mean ± SD: 155.1° ± 5.10°, *n* = 18, Fig. 7A–B); pronotal lateral border not widened at posterior angles, width similar or less wide as lateral border of the pronotum anterior to the posterior angles; carinae between lateral margins and pronotal basal foveae strong, parallel to the fine median line. Basal foveae moderately deep, clearly defined throughout except at the basal area, outer basal foveal groove long and deep, reaching the posterior margin of the pronotum, inner basal foveal groove short and weakly incised, not reaching the posterior margin of the pronotum; basal foveae slightly rugose and sparsely punctate; disc moderately convex and smooth, only very finely and sparsely punctate. **Elytra** oviform (EL/EW = 1.35–1.56, mean ± SD: 1.43 ± 0.05, *n* = 20; EL/PLt = 2.03–2.31, mean ± SD: 2.21 ± 0.08, *n* = 20; EW/PW = 1.17–1.29, mean ± SD: 1.22 ± 0.03, *n* = 20), widest near the middle; elytral base slightly depressed in the middle; striae deeply impressed, with fine and sparse punctures; parascutellar striae long, apex free, short or connected with first stria; parascutellar pore present on the base of first stria. Third interval generally with 3–6 setigerous pores, situated mostly closer to the second stria (location and number of discal pores variable in some individuals: additional pores occasionally present at the first, second, third and fifth intervals, same specimen may has different discal pore placement on left and right elytron); umbilicate series of pores on the ninth interval, each side composed of 16–20 pores, sparser in the middle, denser anteriorly and posteriorly. Hind wings reduce as leathery wing bud. **Ventral side**: pro- and mesoepisternum sparsely punctate and shallowly rugose; metepisternum with coarse punctures; abdominal sterna glabrous in the middle, with shallow wrinkles laterally; sterna IV and V with sparse coarse punctures and shallow rugosity laterally. **Legs** long and slender; first meso- and metatarsomeres with distinct carina on the outer surface, these occur also near the base of the second tarsomeres; fifth tarsomeres with 2–4 pairs of setae on ventral surface. **Male genitalia**: median lobe of aedeagus bent more than 90 degrees at basal 2/5 (Fig. 2C); in lateral view, ventral margin straight in the middle, apical portion not bent to the ventral side; apical orifice slightly turned to the left; apical lamella long and strongly oblique to the right, length about 1.5 times as it basal width; apex strongly widened in dorsal view, widened with similar angles at both left and right margins (Fig. 2A); apex slightly thickened in lateral view. Right paramere very long and strongly bent, gradually narrowed to apex, apical portion thick, apex sharp (Fig. 2B). **Endophallus** (Fig. 3) extending from the dorsal-left side of aedeagus to ventral side, major parts of endophallus located on the ventral side of the aedeagus, basal portion strongly swollen to the dorsal direction; gonopore (**gp**) located near the basal-ventral direction of the aedeagus, pointing towards the aedeagal base. Six distinct recognizable lobes: left lateral lobe (**ll**) compressed, forming a widening triangular shape towards the base of gp when viewed dorsally, surface with fine scales; left ventral lobe (**lv**) divided into two separate sub-lobes; **lv-1** trochoid, apex positioned towards aedeagal base, base adjacent to rb; **lv-2** very small, situated at about half the height of lv-1; right ventral lobe (**rv**) composed of two sub-lobes: **rv-1** small and compressed, on the base of left-ventral surface of endophallus, close to the aedeagal apex, surface with fine scales; **rv-2** large and oblate, between the base of gp and rv-1, surface with fine scales; dorsal lobe (**dl**) very large and strongly bulging, apex coniform and pointing to the ventral direction. **Female genitalia**: spermatheca with the seminal canal as long as about six times the length of the receptaculum; receptaculum tubiform, with round apex; spermathecal gland very long; the seminal canal inserted at the base of the common oviduct, base of the seminal canal sclerotized (Fig. 5). Stylomere 1 (Fig. 4) with thick setae ventro-apically, stylomere 2 with two ensiform setae at the outer margin and with one ensiform seta at the upper middle part of its inner-ventral margin. Tergum VIII (Fig. 5A) with major portion chitinized, two small semi-chitinized patches with dense spots on each side; anterior margin with a wide, U-shaped notch in middle. Sternum VIII (Fig. 5B) with sparse setae on posterior margin; posterior margin curved, deeply notched in the center; posterior region chitinized, anterior region semi-chitinized, with a V-shaped transparent region on the center, adjacent to the central posterior notch.

#### Distribution.

This species is known only from the type locality, Taipinggou Forest Farm, Zhangguangcai Mountain in Heilongjiang Province of China.

#### Etymology.

The name “*silvestris*” derives from the Latin adjective “*silvestris*”, which means “pertaining to a forest or wood”, as well as “living in forest”. This species is named for its distinct habitat, with all individuals collected in natural forest types such as mixed secondary forest and mature forest habitats.

### Pterostichus (Feroperis) maryseae

Taxon classificationAnimaliaColeopteraCarabidae

Sun & Shi sp. n.

http://zoobank.org/F019330F-BD6C-4368-8330-83E992D85A53

**Figures 8–10.**
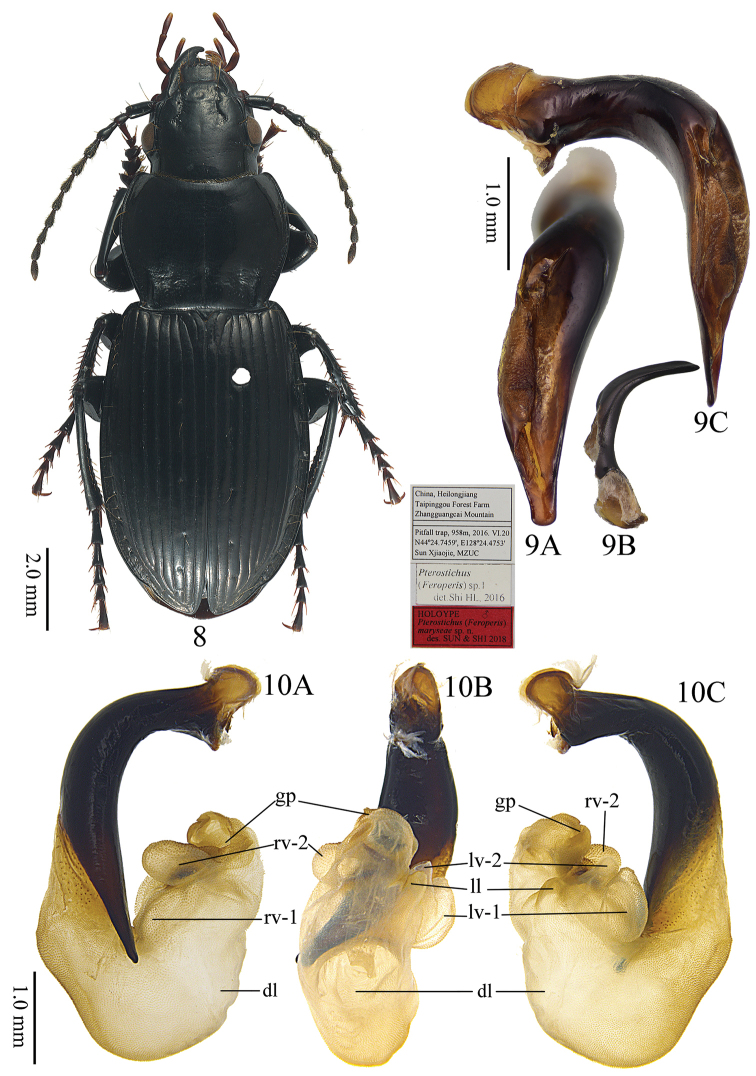
Pterostichus (Feroperis) maryseae sp. n. **8** Habitus of holotype **9** Male genitalia of holotype **A** dorsal view of median lobe, **B** right paramere, **C** left lateral view of median lobe **10** Endophallus of a paratype **A** right lateral view, **B** ventral view, **C** left lateral view.

**Figures 11–13.**
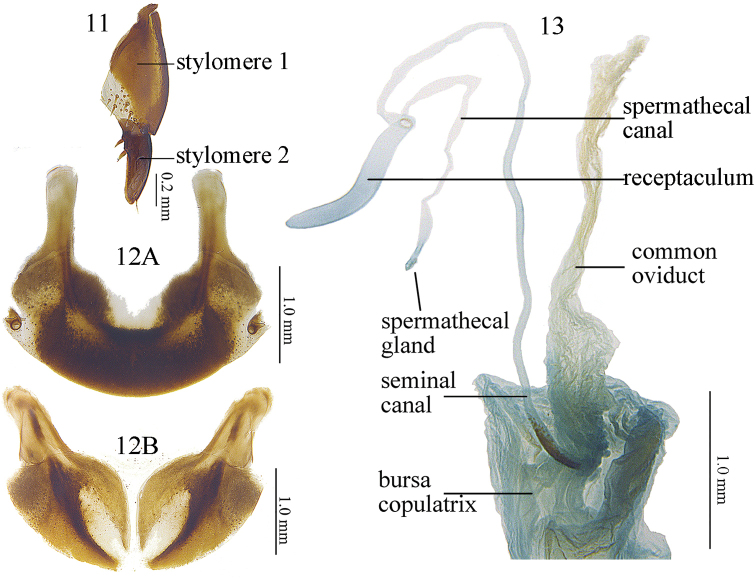
Pterostichus (Feroperis) maryseae sp. n., a female paratype **11** Stylomere of female ovipositor, ventral view **12 A** tergum VIII, **B** sternum VIII **13** Female reproductive tracts.

Figs 7 , 8–10 , 11–13

#### Type locality.

CHINA: Heilongjiang Province, Hailin County: Taipinggou Forest Farm (44°24.7459'N, 128°24.4753'E), altitude 958 m.

#### Type materials.

**Holotype** (IZAS): male, body length 13.4 mm, board mounted, genitalia dissected and glued on plastic film pinned under specimen, “China, Heilongjiang / Taipinggou Forest Farm / Zhangguangcai Mountain”; “Pitfall trap, 958 m, 2016.VI.20 / 44°24.7459'N, 128°24.4753'E / Sun Xiaojie, MZUC”; “HOLOTYPE ♂ / *Pterostichus* (*Feroperis*) / *maryseae* sp. n. / des. SUN & SHI 2018” [red label]. Paratypes (a total of 942 specimens [440 males and 502 females], all in IZAS): 67 males, 162 females, the same data as holotype, but labeled as paratype. 96 males and 97 females, “China, Heilongjiang / Taipinggou Forest Farm / Zhangguangcai Mountain”; “Pitfall trap, 958 m, 2016.VI.08 / 44°24.7459'N, 128°24.4753'E / Sun Xiaojie, MZUC”; “PARATYPE / *Pterostichus* (*Feroperis*) / *maryseae* sp. n. / des. SUN & SHI 2018” [red label]. 89 males and 160 females, “China, Heilongjiang / Taipinggou Forest Farm / Zhangguangcai Mountain”; “Pitfall trap, 958 m, 2016.VII.05 / 44°24.7459'N, 128°24.4753'E / Sun Xiaojie, MZUC”; “PARATYPE / *Pterostichus* (*Feroperis*) / *maryseae* sp. n. / des. SUN & SHI 2018” [red label]. 167 males and 46 females, “China, Heilongjiang / Taipinggou Forest Farm / Zhangguangcai Mountain”; “Pitfall trap, 958 m, 2016. VIII.03 / 44°24.7459'N, 128°24.4753'E / Sun Xiaojie, MZUC”; “PARATYPE / *Pterostichus* (*Feroperis*) / *maryseae* sp. n. / des. SUN & SHI 2018” [red label]. 21 males and 36 females, “China, Heilongjiang / Taipinggou Forest Farm / Zhangguangcai Mountain”; “Pitfall trap, 958 m, 2016.VIII.31 / 44°24.7459'N, 128°24.4753'E / Sun Xiaojie, MZUC”; “PARATYPE / *Pterostichus* (*Feroperis*) / *maryseae* sp. n. / des. SUN & SHI 2018” [red label]. 1 female, “China, Jilin, Jiaohe City / Forest Ecology Stations”; “Pitfall trap, 397 m, 2018.IX.01 / 43°57'20"N, 127°41'50"E / Shi Hongliang, Beijing Forestry University”; “PARATYPE / *Pterostichus* (*Feroperis*) / *maryseae* sp. n. / des. SUN & SHI 2018” [red label].

#### Diagnosis.

This new species can be distinguished from all the other species in the subgenus by the combination of the following characters: (1) lateral margins of pronotum evenly convex before basal third, then strongly contracted and straight before posterior angles; (2) pronotum posterior angles strongly protruding, forming strong denticles, lateral border strongly widened at posterior denticles, its width about two times wider than the lateral border of pronotum; (3) apical lamella of aedeagus about quadrate, length approx 1.1 times its basal width; slightly widened forming truncate apex, not thickened in lateral view; apical lamella weakly bent to the right in dorsal view.

This new species is distinguishable in the subgenus for its apical lamella of the aedeagus not being capitate or widened to apex, and ventral margin straight before apex in lateral view. These aedeagal characters can distinguish it from most species of *Feroperis* except these following six species and subspecies: *P.procaxprocax* Morawitz, 1862, *P.procaxdecastriensis* Lafer, 1979, *P.shingarevimaichensis* Lafer, 1979, *P.shingarevishingarevi* Lafer, 1979, *P.arsenjevi* Lafer, 1979, *P.odaesanensis* Lafer, 2011. Besides *P.shingarevishingarevi*, all other five taxa are different from the new species by the pronotum posterior angle being rounded, obtuse or only weakly dentate, but the lateral border never widened at posterior denticles. Therefore, *P.shingarevishingarevi* (Primorsky Krai: Evseevka, 44°24'N, 132°52'E) is considered to be the most similar species to *P.maryseae* sp. n.

When compared with *P.shingavrevishingavrevi* Lafer, 1979, *P.maryseae* sp. n. can be differentiated by: (1) in *P.maryseae*, the pronotum being widest at about basal 2/3, while it is widest near the middle in *P.shingavrevi*; (2) in *P.maryseae*, the apical lamella of aedeagus more obviously truncate, its left margin abruptly bent at about apical third of the apical lamella, while in *P.shingavrevi*, the apical lamella is less truncate, its left margin slightly bent near the middle of the apical lamella.

*P.maryseae* sp. n. is sympatric to the second new species, *P.silvestris*. These two new species can be readily distinguished by their differences in their pronotal posterior angles: in *P.silvestris*, the posterior angles of the pronotum are weakly protruding and dentate, lateral border not widened at the posterior denticles, its width similar to or less than the lateral border of the pronotum; in *P.maryseae*, posterior angles of the pronotum are strongly protruding and dentate, lateral border distinctly widened at the posterior denticles, its width at least twice as wide as the lateral border of the pronotum. They also strongly differ in their male genitalia (Figs 2, 9): apical lamella of aedeagus much longer and apex distinctly widened in *P.silvestris*; endophallus with a large coniform dorsal lobe in *P.silvestris*, with such a lobe being absent in *P.maryseae*. Moreover, these two species are also different in their female genitalia: the female reproductive tract with seminal canal shorter in *P.maryseae*, about four times length as the receptaculum (versus six times length as the receptaculum in *P.silvestris*); sternum VIII with the V-shaped transparent region shorter and wider in *P.maryseae*.

#### Description.

Body length 12.6–14.9 mm (mean ± SD: 13.5 ± 0.56, *n* = 20), both sexes with similar body shape. Dorsal surface black and shiny; head and pronotum without obvious microsulpture; elytra with very fine and isodiametric microsculpture. **Head** mostly smooth, frons and vertex shiny, with scattered micro-punctures; eyes moderately convex; antennae just reaching the pronotum base. **Pronotum** approximately 1.4 times wider than head (PW/HW =1.26–1.52, mean ± SD: 1.40 ± 0.05, *n* = 20); rounded in shape, widest at about 2/3 length to the posterior margin (PW/PLt = 1.21–1.36, mean ± SD: 1.28 ± 0.04, *n* = 20; PW/PLm = 1.37–1.59, mean ± SD: 1.46 ± 0.05, *n* = 20); lateral margins evenly convex from apex to about basal 1/3, then strongly contracted and almost straight before the posterior angles (PW/PA = 1.29–1.48, mean ± SD: 1.36 ± 0.05, *n* = 20; PW/PB = 1.29–1.43, mean ± SD: 1.36 ± 0.04, *n* = 20); apical width of pronotum nearly same as its basal width (PB/PA = 0.93–1.07, mean ± SD: 1.00 ± 0.04, *n* = 20). Anterior angles obtuse and rounded, distinctly contracted inward; lateral channels narrow in front of midpoint and gradually expanded towards the base, with flatten and sparse punctures on them. Posterior angles strongly protruding, forming strong denticles, lateral border at the posterior denticles strongly widened, at least twice as wide as the lateral broder of the pronotum anterior to the posterior angles; lateral border interrupted before posterior denticles; the posterior denticles about 90° (Fig. 7C–D), commonly with a side edge (128.6°–152.6°, mean ± SD: 140.7° ± 8.98°, *n* = 10); carinae between lateral margins and pronotal basal foveae clearly defined, parallel to the median line. Basal foveae moderately deep, clearly defined throughout except at the basal area, outer basal foveal groove long and deep, reaching the posterior margin of pronotum, inner basal foveal groove short and weakly incised, base separated from the posterior margin; basal foveae slightly rugose and sparsely punctate; disc moderately convex and smooth, only very finely and sparsely punctate. **Elytra** oviform (EL/EW = 1.30–1.47, mean ± SD: 1.39 ± 0.05, *n* = 20; EL/PLt = 2.09–2.33, mean ± SD: 2.17 ± 0.07, *n* = 20, EW/PW = 1.16–1.30, mean ± SD: 1.21 ± 0.03, *n* = 20), widest near the middle; elytra base slightly depressed in the middle; striae deeply impressed, with fine and sparse punctures; parascutellar stria long, apex free, short and incomplete or connected with first stria, normally located between the first stria and elytra suture, occasionally between first and second stria; parascutellar pore present on the base of first stria. Third interval generally with 3–6 setigerous pores, situated mostly closer to the second stria, occasionally 1–2 additional pores may present on the first and fifth intervals; umbilicate series of pores on the ninth interval, each side composed of 16–20 pores, sparser in the middle and denser anteriorly and posteriorly. Hind wings strongly vestigial, only developed as leathery wing bud. **Ventral side**: pro- and mesoepisternum sparsely punctate and shallowly rugose; metepisternum with coarse punctures; abdominal sterna glabrous in the middle, with sparse coarse punctures laterally; lateral area of sterna IV and V densely rugose. **Legs** long and slender; first meso- and metatarsomeres with distinct carina on the outer surface, these occur also near the base of the second tarsomeres; fifth tarsomere with 2–4 pairs of setae on ventral surface. **Male genitalia**: median lobe of male genitalia bent more than 90 degrees at basal 2/5 (Fig. 9C); in lateral view, ventral margin almost straight in the middle, apical portion not bent to the ventral side, apical lamella slightly depressed from dorsal to ventral side; on dorsal view (Fig. 9A); apical orifice slightly turned to the left; apical lamella sub-quadrate, slightly narrowed to apex, about 1.1 times as its basal width, slightly oblique to the right; apex a little truncate, left margin of the apical lamella abruptly bent at about apical 1/3. Right paramere very long and strongly bent, a little narrowed to apex, apical portion thick, apex obliquely truncate (Fig. 9B). **Endophallus** (Fig. 10) extending from the dorsal-left side of aedeagus to ventral side, major parts of the endophallus located on the ventral side of the aedeagus, basal portion slightly swollen to the dorsal direction; gonopore (**gp**) located near the basal-ventral direction of the aedeagus, pointing towards the aedeagal base. Five distinct recognizable lobes: left lateral lobe (**ll**) compressed, forming a widening triangular shape towards the base of gp when viewed dorsally, surface with fine scales; left ventral lobe (**lv**) divided into two separate sub-lobes; **lv-1** round, apex positioned towards aedeagal base, base adjacent to rb; **lv-2** very small, situated at about half the height of lv-1; right ventral lobe (**rv**) composed of two sub-lobes: **rv-1** small and compressed, on the base of left-ventral surface of endophallus, close to the aedeagal apex, surface with fine scales; **rv-2** large and round, between the base of gp and rv-1, surface with fine scales; dorsal lobe absent. **Female genitalia**: spermatheca with the seminal canal as long as about four times the length of the receptaculum; receptaculum tubiform, apical slightly pointed; spermathecal gland long; the seminal canal inserted at the base of the common oviduct, base of the seminal canal sclerotized (Fig. 13). Stylomere 1 (Fig. 11) with thick setae ventro-apically, stylomere 2 with two ensiform setae at the basal half of outer margin and with one ensiform seta at the upper middle part of its inner-ventral margin. Tergum VIII (Fig. 12A) with major portion chitinized, two small semi-chitinized patches with dense spots on each side; anterior margin with large quadrate middle notch. Sternum VIII (Fig. 12B) with sparse seta the on posterior margin; posterior margin curved, deeply notched in the center; posterior region chitinized, anterior region semi-chitinized, with a V-shaped transparent region on the center, shorter and wider than the previous species, adjacent to the central posterior notch.

#### Distribution.

This species is only known form the Zhangguangcai Mountain range on the border of Jilin and Heilongjiang Provinces of China. Two localities in the Hailin County of Heilongjiang Province and Jiaohe County of Jilin Province were recorded.

#### Etymology.

This species is named after Miss Maryse Diekman, who collected many specimens of both new species.

### Pterostichus (Feroperis) acutidens

Taxon classificationAnimaliaColeopteraCarabidae

(Fairmaire, 1889)

Fig. 14A

Omaseus acutidens Fairmaire, 1889: cc (original: Omaseus; syntype in Muséum National d’Histoire Naturelle, Paris, France; type locality: “Pékin”); Jedlička 1962: 251; Lafer 1979: 8.

#### Type locality.

Beijing.

#### Type material examined.

Syntype of *Omaseusacutidens* Fairmaire, 1 male (MNHN), “*Omaseus / acutidens / Fairm. / Pekin*”; “SYNTYPE” [red label]; “*Omaseus* / *acutidens* / *Fairmaire* / Det. Shi H.L. 2011”; “Muséum Paris / 1906; Coll. Léon Fairmaire”.

#### Other material examined.

7 specimens (IZAS), “China, Hebei Province, Xinglong County, Wuling Mountain. 1994.V.23, YU Peiyu”; 5 specimens (IZAS), “China, Beijing, Huairou District, Yunmeng Mountain. 800 m, 2005.VII.13, LIU Ye”; 628 specimens (IZAS), “China, Beijing, Dongling Mountain. Pit fall trap, 1160–1410 m, 2011.VI.10–2012.IX.13, ZOU Yi”.

**Figure 14.**
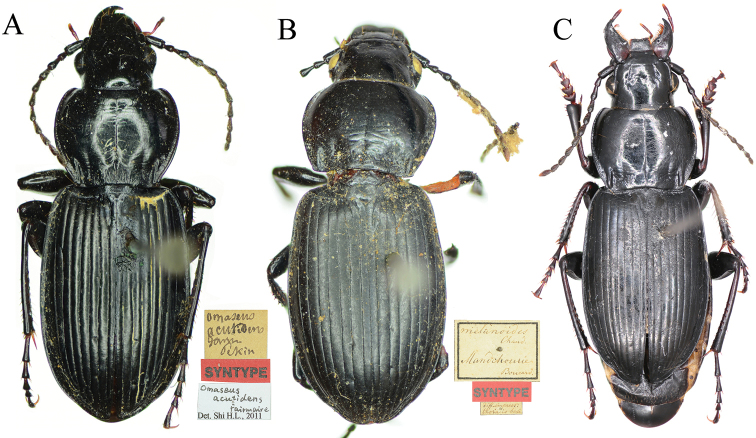
Habitus of Pterostichus (Feroperis) spp. from China. **A***P.acutidens* (Fairmaire), male syntype (MNHN) **B***P.melanodes* (Chaudoir), female syntype (MNHN) **C***P.rasilis* Park & Kwon, male from Changbai Mountain (IZAS).

#### Diagnosis.

Body length 14–17 mm, blackish, elytra shiny without metallic lustre. Pronotum subcordate, widest at approximately anterior 2/5; lateral margins of pronotum strongly constricted to the base; posterior angles strongly protruding and forming very strong denticles, lateral border at the posterior denticles strongly widened, at least twice as wide as the lateral broder anterior to the posterior angles; lateral border not interrupted before posterior denticles; basal foveae with a few large punctures. Elytra with humeral teeth faint; the third interval usually with 3–4 setigerous pores close to the second stria, but position variable. Apical lamella of aedeagus approximately triangular, gradually narrowed to apex, apex not widened or thickened; in lateral view, apical lamella distinctly bent downwards. Right paramere long and bent, apex distinctly compressed.

This species can be distinguished from all the other species of the subgenus by its male genitalia: aedeagus with apical lamella simple and distinctly bent downwards. From the external appearance, this species is superficially similar to *P.jungens* Tschitschérine, 1893; they can be distinguished by: the pronotal lateral channel before posterior angles wider in *P.acutidens* than in *P.jungens*; and quite different shape of apical lamella of aedeagus.

#### Distribution.

This species is only known from Beijing and adjacent regions in the north part of Hebei Province.

### Pterostichus (Feroperis) melanodes

Taxon classificationAnimaliaColeopteraCarabidae

(Chaudoir, 1878)

Fig. 14B

Feroperis melanodes Chaudoir, 1878: 69 (original: Feronia; syntype in Muséum National d’Histoire Naturelle, Paris, France; type locality: “Mandchourie”). Jedlička 1962: 249; Lafer 1979: 29.

#### Type locality.

Manchuria, without reference to the exact type locality.

#### Type material examined.

Syntype of *Feroniamelanodes* Chaudoir, 1 female (MNHN), “*melanoides / Chaud. / Manchourie / Bouchard*”; “SYNTYPE” [red label]; “*Diffimpress / thoracis basi*”.

#### Diagnosis.

Body length about 15 mm, blackish, elytra shiny without metallic lustre. Pronotum round, widest at approximately anterior 1/3; lateral margins of pronotum strongly constricted to the base; lateral margins straight before posterior angles; posterior angles obtuse, with indistinct small denticles, lateral border at the posterior denticles less widened than the lateral broder anterior to the posterior angles; basal foveae rugose and convex, without punctures; basal foveae faintly defined, outer basal foveal groove short, inner basal foveal groove invisible, carinae between basal foveae and lateral margin shallower than other species. Elytra without humeral teeth; the third interval usually with 3 setigerous pores close to the second stria. Fifth tarsomere of all legs setose on ventral side. Male genitalia unknown.

#### Distribution.

Only known by the type materials from “Manchuria”, referring to the northeastern Provinces of China, without specified exact locality.

#### Remark..

*P.melanodes* was described on a female specimen from “Manchuria” that refers to a large area including Provinces Liaoning, Jilin, Heilongjiang and eastern parts of Inner Mongolia of present administrative divisions in China. While the exact type locality remains unspecified, *P.melanodes* is one of the earliest described, but also least known species in the subgenus, because neither its male genitalia nor the exact type locality are known. Lafer (1979) included *P.melanodes* as a dubious species in his work on *Feroperis* and extensively discussed its possible distribution and taxonomical position. Compared with other species from China and Russia, the species in question appears most similar to *P.sungariensis* Lafer, 1979 and *P.chechcirensis* Lafer, 1979 for their similar pronotum shape. Further distinctions between these species remain problematic as long as the male genitalia of *P.melanodes* remain unknown.

### Pterostichus (Feroperis) rasilis

Taxon classificationAnimaliaColeopteraCarabidae

Park & Kwon, 1996

Fig. 14C

Pterostichus (Feroperis) rasilis Park & Kwon, 1996: 3 (holotype in Systematic Entomology Laboratory, Department of Agricultural Biology, Kyungpook National University, Republic of Korea; type locality: northern slopes of Changbai Mountain, Jilin, China); Lafer 2011: 434.

#### Type locality.

Northern slopes of Changbai Mountain, Jilin, China.

#### Material examined.

46 specimens (IZAS),“China, Jilin, Changbaishan Nature Reserve; 2011.VII.14, 42°3'15"N, 128°4'2"E–42°10'47"N, 128°8'15"E, 870–2000 m, Zou Yi”; 83 specimens (IZAS),“China, Jilin, Changbaishan Nature Reserve; 2011.VII.27, 42°3'15"N, 128°4'2"E–42°7'16"N, 128°6'27"E, 1330–2000 m, Zou Yi”; 39 specimens (IZAS),“China, Jilin, Changbaishan Nature Reserve; 2011.VIII.08, 42°3'15"N, 128°4'2"E–42°7'15"N, 128°6'26"E, 1330–2000 m, Zou Yi”; 30 specimens (IZAS),“China, Jilin, Changbaishan Nature Reserve; 2012.VII.14, 42°3'15"N, 128°4'2"E–42°5'41"N, 128°4'3"E, 1520–2000 m, Zou Yi”; 94 specimens (IZAS),“China, Jilin, Changbaishan Nature Reserve; 2012.VII.12, 42°3'15"N, 128°4'2"E–42°7'9"N, 128°6'17"E, 1350–2000 m, Zou Yi”; 62 specimens (IZAS),“China, Jilin, Changbaishan Nature Reserve; 2012.VII.30, 42°3'15"N, 128°4'2"E–42°7'9"N, 128°6'17"E, 1330–2000 m, Zou Yi”.

#### Diagnosis.

Body length 13–15 mm, blackish, elytra shiny without metallic lustre. Pronotum subcordate, widest at approximately anterior 1/3; lateral margins of pronotum strongly constricted to the base; posterior angles strongly protruding and forming very prominent denticles, lateral border at the posterior denticles strongly widened, at least twice as wide as the lateral broder of the pronotum anterior to the posterior angles; lateral border interrupted before posterior denticles; basal foveae rugose. Elytra with faint humeral teeth; the third interval usually with 3 or 4 setigerous pores close to the second stria, but position variable. Apical lamella of aedeagus elongate, apex slightly widened at both left and right margins in dorsal view; not thickened or bent downwards in lateral view. Right paramere long and bent, gradually narrowed to apex, apex pointed.

This species can be distinguished from most species of the subgenus by: male genitalia with apical lamella of aedeagus rectangular, apex widened in dorsal view, but not thickened in lateral view, and pronotal posterior angles with strong denticles. From the above characters, this species is superficially similar to *P.vladivostokensis* Lafer. They can be distinguished by: in *P.rasilis*, the apical lamella of aedeagus shorter, length approximate 1.2 times as basal width, apex less widened, not capitate; while in *P.vladivostokensis* the apical lamella of aedeagus longer, length approximate 1.5 times as basal width, apex strongly widened, distinctly capitate.

#### Distribution.

This species is known only from the northern slopes of Changbai Mountain, Jilin province in China, and it is a locally abundant species. It is probably also distributed within the DPR Korea, on the eastern slopes of Paektusan (the Korean name of Changbai Mountain).

#### Remark.

This species was originally described based on 14 specimens from Paektusan, Hamgyeongbuk-do, DPR Korea. Lafer (2011) examined the type specimens and specified that the true type locality was on the northern slopes of the Changbai Mountain in the territory of China, based on a personal communication with Y.J. Kwon in 1994.

## Discussion

The two new species described here conform to the commonly observed trend for relatively small geographic distribution ranges in Chinese members of the genus *Pterostichus*, forming two sibling species that are very similar in their external morphology, but that strongly differ in the structure of their genitalia. These two new species were initially difficult to identify, particularly with regards to some female specimens that are superficially similar in their external morphological features, but the male genitalia can readily distinguish them as distinct species. Based on the morphological features, we subsequently ascertained their identity based on the species’ distribution patterns. It appears that *P.maryseae* occurs in plantation forests and secondary poplar-dominated humid forests. While *P.silvestris* is also encountered in secondary poplar-dominated forests, its main distribution appears to be in secondary mixed forests and remnants of mature forest that have persisted in the large-scale deforestation campaigns across temperate eastern China before the middle of the last century. This pattern highlights the need for detailed ecological information to be provided when collecting specimens, which will greatly facilitate their subsequent identification.

A total of 2587 specimens of the two new species in 80 pitfall trap sample plots were collected in the present study. Using a stereoscope to examine every specimen and dissecting genitalia of 56 male and 48 female specimens, we established that Pterostichus (Feroperis) silvestris accounted for 737 female and 907 male individuals, while Pterostichus (Feroperis) maryseae accounted for 502 female and 441 male specimens. Based on in-depth investigations of specimens representing the new species, we found that the number of setigerous pores and their location on the interval of the elytron are highly variable. The exceptions were pores on the third elytral interval, where we commonly encountered 3 or 4 setigerous pores adjacent to the second stria. The number of setigerous pores and their location has been described in great detail for some species in *Feroperis* (i.e. Park and Kwon 1996, Lafer 2011). In our view, the structure of pronotum, the apical lamella of median lobe, and the geographic distribution represent the most important features to determine the different species in *Feroperis*, which was also stressed by Lafer (1979).

## Supplementary Material

XML Treatment for Feroperis

XML Treatment for Pterostichus (Feroperis) silvestris

XML Treatment for Pterostichus (Feroperis) maryseae

XML Treatment for Pterostichus (Feroperis) acutidens

XML Treatment for Pterostichus (Feroperis) melanodes

XML Treatment for Pterostichus (Feroperis) rasilis
